# Effects of a weight-gain restriction programme for obese pregnant women on sickness absence and pregnancy benefits

**DOI:** 10.3109/02813432.2012.754091

**Published:** 2013-06

**Authors:** Gunilla Sydsjö, Wiktor Gustafsson Monfils, Nicholas de Keyser, Ing-Marie Claesson, Adam Sydsjö, Ann Josefsson

**Affiliations:** Division of Obstetrics and Gynaecology, Department of Clinical and Experimental Medicine, Faculty of Health Sciences, Linköping University, Department of Obstetrics and Gynaecology in Linköping, County Council of Östergötland, Linköping, Sweden

**Keywords:** General practice, obesity, pregnancy, pregnancy benefit, sickness absence, Sweden, weight restriction

## Abstract

**Objective:**

To evaluate the effect of a weight-gain restriction programme for obese pregnant women on sickness absence days and pregnancy benefit days during pregnancy and postpartum.

**Design:**

A prospective, controlled intervention study. The Swedish Social Security Agency's records were utilized to compile sickness absence and pregnancy benefit information.

**Setting:**

Antenatal care clinics in the south-east of Sweden.

**Subjects:**

One hundred fifty-five obese pregnant women who participated in a weight restriction program with weekly structured motivational and behavioural talks combined with aqua-aerobics during pregnancy. A total of 193 obese pregnant women with no intervention served as controls.

**Main outcome measures:**

Sickness absence benefits and pregnancy benefits expressed as a percentage.

**Results:**

On average women in the intervention group had 76.68 total full days of sickness absence benefit compared with 53.09days in the control group. Total full days of pregnancy benefits were 39.66% days and 41.41% for the intervention and control groups respectively. For the women who were on sick leave there were no differences between the groups in the amount of days taken.

**Conclusions:**

Given the complexity of factors that have an influence on sickness absence leave, it is possible that programmes that do not address the influence of social aspects and attitudes towards sickness absence have limited effect.

- The proportion of overweight women of child-bearing age has tripled, and obesity in that population has increased fivefold in Sweden over the last three decades.
- The weight-gain restriction programme during pregnancy for obese women did not have any impact on their level of sick leave.
- Pregnancy benefits were equally distributed between the two groups.

## Introduction

The estimated direct medical costs stemming from overweight/obesity in Sweden are roughly 2% of the health care budget – amounting to €300 million. Indirect costs such as sickness benefits and loss of production are more difficult to calculate, but are estimated to be similar, total costs being roughly €600 million for a country with a population of 9 million [1,2]. Obesity is not confined to a single subset of population but is spread throughout all ethnicities, age groups, sexes, and socio-economic classes and, of interest here, pregnant women [2–4]. A Swedish study indicates that some of these risks can be averted if a woman's pregnancy weight gain remains under 8 kg [5]. The number of intervention studies to control excessive pregnancy weight gain is growing and show divergent results [6–13]. The proportion of overweight women of child-bearing age has tripled, and obesity in that population has increased fivefold in Sweden over the last three decades [14]. It would therefore be reasonable to assume that this trend translates to increased direct and indirect medical costs.

A Swedish pregnant woman has generous access to a variety of social benefits. Since 1980, Pregnancy Benefit is obtainable for women with arduous work with 50 days of paid leave, which may start as early as 60 days before the due date [15]. The Swedish social security agency does not classify pregnant women as a sub-population in its presentation of statistics, so present levels of sickness absence benefits during pregnancy are not known. However, studies from the late 1990s showed that pregnant women comprised 22% of all sick-listed women in the age group 16 to 44, and approximately every second woman received sickness absence benefits during her pregnancy [16,17]. Furthermore, it has been postulated by the Swedish social security agency, based on those studies, that these proportions remain roughly the same [16,17].

In an intervention programme for obese pregnant women we found that it is possible to control weight gain to < 7 kg [18] with motivational meetings based on a cognitive behavioural strategy [19]. The purpose of this study is to evaluate whether this weight reduction also has an impact on sickness absence days and pregnancy benefit days during pregnancy and postpartum.

## Material and methods

### Design of the weight gain restriction programme

Pregnancy weight gain was controlled in the intervention group through 30 minutes of individual weekly motivational meetings with a specially trained midwife. The sessions included weight control and supportive talk. All women who attended the programme were also invited to an aqua aerobics class (once or twice a week), especially designed for obese women. The target weight gain limit was < 7 kg. The proportion of women who gained less than 7 kg was higher (p = 0.003) in the intervention group (35.7%) compared with the control group (20.5%). There was a significant difference in socio-economic groups (p = 0.044) but not in occupational status between the two groups [18]. For a detailed description of the study design, intervention, and background characteristics of the participating women, see Claesson et al. [18,20].

The Swedish antenatal health care system reaches almost 100% of all pregnant women. The antenatal and delivery care is free of charge.

### Intervention group

All obese (BMI > 30, n = 317) pregnant women consecutively registered during two years at the ANCs in Linköping were approached. Exclusion criteria: inability to understand Swedish, a pre-pregnant diagnosis of diabetes, thyroid dysfunction, or a psychiatric disease treated with neuroleptic drugs excluded 45 women. Thirteen women had a miscarriage or a legal abortion and were also excluded as well as 29 women who moved out of the catchment area.

In all, 230 obese women were thus eligible and invited to participate. Of these, 70 women refrained from participation and five women dropped out during the intervention. A total of 155 obese women (67.4%) accepted and completed the intervention.

### Control group

To constitute a control group, all obese, pregnant women (BMI > 30, n = 437) consecutively registered during the same period at the ANCs in two nearby cities were approached. The exclusion criteria were the same as for the index women and 42 women were excluded. Ten women had an early miscarriage or a legal abortion. In all, 385 were invited to participate; 177 women refrained and 15 women dropped out. Finally, 193 women accepted and completed participation (50.1%). The obese women in the control group attended the routine antenatal care programme.

The following data were collected at the women's first visit at the ANC: age, parity, marital status, occupation, and smoking habits. The women's weight was registered at the first visit to the ANC, during the pregnancy, and at the postnatal check-up.

### Sickness absence

Information on sickness absence benefits during pregnancy, and up to eight weeks post-partum, was obtained from the Swedish Social Security Agency. The duration of pregnancy was determined by ultrasound in early pregnancy and recorded in the patient's record. Available information in the archive included benefits paid to employed women after the mandatory 14-day period during which the employer pays the benefit, and benefits paid to unemployed women after a mandatory one-day unpaid period. Information on benefits paid to employed women during the first 14 days of illness is not registered in the archive. Sick-leave benefits can be adjusted to 25%, 50%, 75%, or 100%. To calculate each individual's total paid sick-leave days, the total number of days of 25% benefits was multiplied by 0.25 and added to the other values as described in the formula:

Days 25% benefit* 0.25

Days 50% benefit* 0.5

Days 75% benefit* 0.75

+ Days 100% benefit

Total full days of sickness absence benefit

### Pregnancy benefit

This benefit is also adjustable to the 25%, 50%, 75%, or 100% levels. The total full days of pregnancy benefit was calculated in the same way as total full days of sickness absence benefit.

### Sub-group analyses

For sub-group analyses within the intervention group we first analysed the group of women who held their weight-gain to under 7 kg. Weight gain was calculated using the last registered weight during motivational meeting minus weight at enrolment in the programme. We also did a sub-group analysis on the women in the intervention group on the correlation between enrolment BMI, benefits, and weight gain.

### Statistics

The chi-squared test was used to compare the variables included in background characteristics. Student's t-test was used to compare means of duration of pregnancy, total days of sickness absence, and total days of pregnancy benefit variables between the intervention and control groups as well as between each respective sub-group and the control group.

## Results

There was no difference in gestational length between the two study groups. Mean duration of gestational length was 39.32 and 39.34 weeks for the intervention and control groups, respectively (p = 0.93). No significant differences in background characteristics between intervention and control groups were found (Table I).

**Table I.** Comparison of background characteristics for the obese pregnant women.

	Intervention (n = 155) %	Control (n = 193) %	p-value*
Employed at enrolment	64.5	70.5	0.12
Family situation:			0.68
Couple	93.5	91.9	
Single	2.6	4.1	
Other	3.9	3.5	
Not stated	0.0	0.6	
Smoking:			0.88
No	91.6	92.4	
1–9 per day	5.2	5.2	
> 9 per day	3.2	2.3	
Alcohol consumption:			0.26
Never/very seldom	99.4	97.7	
At most once per week	0.0	1.7	
More than once per week	0.0	0.0	
Not reported	0.6	0.6	

*Chi-squared test.

The percentage of women in the intervention group who received registered sickness absence benefits was 29.0% compared with 34.7% in the control group (p = 0.26). Pregnancy benefits were 31.6% and 34.2% for the intervention and control groups respectively (p = 0.61). Furthermore, no differences were found when considering sickness absence and pregnancy benefits for only those women who were employed at the beginning of the programme. The total days of both sickness absence benefit and pregnancy benefit did not differ between the groups (Table II).

**Table II.** Mean number of days of sickness absence benefit and pregnancy benefit for the obese pregnant women.

	Intervention (n = 155) mean (SD)	Control (n = 193) mean (SD)	p-value*
Sick-leave benefit:			
25%	1.90 (13.45)	1.03 (8.00)	0.46
50%	4.08 (18.09)	5.03 (20.03)	0.65
75%	0.37 (3.34)	1.36 (7.76)	0.11
100%	19.47 (53.73)	14.74 (38.39)	0.36
Total full days of sickness absence benefit	22.26 (56.00)	18.53 (42.09)	0.48
Total full days of sickness absence benefit†	76.68 (81.89)	53.09 (57.36)	0.1
Pregnancy benefit:			
25%	0.00 (0.00)	0.00 (0.00)	‡
50%	2.24 (9.53)	1.54 (7.63)	0.45
75%	0.00 (0.00)	1.14 (7.75)	0.04
100%	11.42 (20.16)	12.53 (20.85)	0.62
Total full days of pregnancy benefit	12.54 (20.08)	14.16 (21.72)	0.48
Total full days of pregnancy benefit†	39.66(13.99)	41.41(15.73)	0.54

*Student's t-test. †Calculated for those women who were employed at start of programme. ‡p-value not calculated.

The total number of days of benefits in all sub-groups in the intervention and control groups (i.e. < 7 kg intervention compared with < 7 kg control) showed no significant differences.

We also did a sub-group analysis on the women in the intervention group on weight gain, enrolment BMI 30–35 and > 35 and found no difference in total benefits days and sick-leave as well as pregnancy benefit and found no differences (Tables III–IV).

**Table III.** Pearson correlations: Enrolment BMI and benefits; gestational weight gain and benefits for the women in the intervention group.

	BMI (n = 155) (p-value)	Weight gain (n = 155) (p-value)
Sick-leave benefit	0.15 (0.06)	–0.05 (0.55)
Pregnancy benefit	0.01 (0.87)	–0.02 (0.78)

**Table IV.** Subgroups: Top one-third gestational weight gain and bottom one-third weight gain as well as enrolment BMI 30–35 and > 35 for the women in the intervention group.

	Top one-third (n = 50) mean (SD)	Bottom one-third (n = 52) mean (SD)	p-value*	BMI 30–35 (n = 101) mean (SD)	BMI > 35 (n = 54) mean (SD)	p-value*
Total sick-leave benefit	24.0 (60.0)	22.5 (56.6)	0.90	17.4 (45.7)	31.4 (71.0)	0.19
Total pregnancy benefit	11.1 (19.2)	13.1 (20.8)	0.62	12.9 (20.4)	11.8 (19.6)	0.75

*Chi-squared test.

## Discussion

The weight-gain restriction programme during pregnancy for obese women did not have any impact on their level of sick leave.

Increasing use and high costs of social benefits highlight the importance of information on possible savings to society after implementation of intervention programmes. Even though this study has several limitations such as a low participation rate in the control group from the start, and exclusion of women who do not speak and read Swedish, it is the first analysis of a weight-gain restriction intervention programme during pregnancy for obese women, and its effect on paid sickness absence benefits. The present sick-leave rate among Swedish women is not known since the Swedish Social Security Department does not distinguish between pregnant and non-pregnant women in its statistics. However, studies on the rate of sickness absence leave for pregnant women were conducted in Sweden during the 1980s and 1990s up to and including 1997 when the rate was 53% [17]. Since our post-14-day results show that roughly 20% of pregnant women receive sickness benefits, the pre-14-day proportion is supposedly quite large. However, since there was no difference in level of employment between the groups at the time of enrolment, it can be projected that any effect of the programme on sickness absence benefits during the first 14 days would mirror the results starting on day 15; that is, no difference. The results, therefore, imply that the intervention programme has no significant effect on a reduction in sickness absence benefits during pregnancy and eight weeks post-partum.

No changes in the rate of pregnancy benefit were expected since a woman's working environment is not affected by the health of the woman. The results supported this assumption. Although no differences in sickness absence benefits or pregnancy benefits were found between sub-groups and control, it is worth noting that in a parallel cost-benefit study we showed that the intervention sub-group that had a pregnancy weight gain of 4.5–9.5 kg showed a mean cost for health care services during pregnancy that was roughly €600 less than the control group's mean [21]. This decrease in cost is due to fewer physician appointments, physician consultations, midwife appointments and fewer days or less cost of hospitalization. That better pregnancy health did not lead to reduced use of sickness absence benefits or the pregnancy benefit may be explained by the possibility that the use of social benefits is motivated to a greater extent by social, economic, and psychological factors than by the actual physical state of these women [22]. We previously studied the effect of the intervention programme on medical costs by comparing the intervention and control groups and discovered no reduced cost during pregnancy, delivery, or the neonatal period for the intervention group as a whole [21]. Coupled with this study's findings, it can be concluded that the intervention programme did not result in any measured medical or social benefit cost reductions. The relationship between physical and mental health and the level of sickness absence is complex, and factors such as acceptance, expectations, and attitude play an important role. In fact, findings by Sydsjö and co-workers (2007) show that levels of sickness absence are strongly correlated with the type of work, and no difference could be shown with respect to sickness absence levels between obese and normal-weight pregnant women [23]. Trends in pregnancy sickness absence have, historically, closely followed the state of the national economy and the level of ensuing benefits suggesting that sickness absence is, in part, economically motivated as opposed to physically motivated [17]. Some 74% of women who received sickness absence benefits during pregnancy reported that their subjectively experienced personal health status during pregnancy was “excellent” or “good” while only 26% reported their health as “bad” or “very bad” [24]. Given the complexity of factors that have an influence on sickness absence leave, it is possible that programmes that do not address the influence of social aspects and attitudes towards sickness absence have limited effect.

## Ethical approval

The study was approved by the Regional Ethical Review Board in Linköping.

## Funding

This study was supported by grants from the Health Research Council of Southeast Sweden (FORSS).
